# A High-Fat Diet Containing Lard Accelerates Prostate Cancer Progression and Reduces Survival Rate in Mice: Possible Contribution of Adipose Tissue-Derived Cytokines

**DOI:** 10.3390/nu7042539

**Published:** 2015-04-09

**Authors:** Han Jin Cho, Gyoo Taik Kwon, Heesook Park, Hyerim Song, Ki Won Lee, Jung-In Kim, Jung Han Yoon Park

**Affiliations:** 1 WCU Biomodulation Major, Department of Agricultural Biotechnology and Center for Food and Bioconvergence, Seoul National University, Seoul 151-921, Korea; E-Mail: hanjini@snu.ac.kr; 2 Department of Food Science and Nutrition, Hallym University, Chuncheon 200-702, Korea; E-Mails: kgt486@hallym.ac.kr (G.T.K.); krecencia@hanmail.net (H.P.); hyerim0715@hallym.ac.kr (H.S.); 3 Advanced Institutes of Convergence Technology, Seoul National University, Suwon 443-270, Korea; 4 Department of Smart Food and Drugs, School of Food and Life Science, Inje University, Gimhae 621-749, Korea; E-Mail: fdsnkiji@inje.ac.kr

**Keywords:** high-fat diet, prostate cancer, survival rate, TRAMP, cytokines

## Abstract

To examine the effects of high-fat diet (HFD) containing lard on prostate cancer development and progression and its underlying mechanisms, transgenic adenocarcinoma mouse prostate (TRAMP) and TRAMP-C2 allograft models, as well as *in vitro* culture models, were employed. In TRAMP mice, HFD feeding increased the incidence of poorly differentiated carcinoma and decreased that of prostatic intraepithelial neoplasia in the dorsolateral lobes of the prostate, which was accompanied by increased expression of proteins associated with proliferation and angiogenesis. HFD feeding also led to increased metastasis and decreased survival rate in TRAMP mice. In the allograft model, HFD increased solid tumor growth, the expression of proteins related to proliferation/angiogenesis, the number of lipid vacuoles in tumor tissues, and levels of several cytokines in serum and adipose tissue. *In vitro* results revealed that adipose tissue-conditioned media from HFD-fed mice stimulated the proliferation and migration of prostate cancer cells and angiogenesis compared to those from control-diet-fed mice. These results indicate that the increase of adipose tissue-derived soluble factors by HFD feeding plays a role in the growth and metastasis of prostate cancer via endocrine and paracrine mechanisms. These results provide evidence that a HFD containing lard increases prostate cancer development and progression, thereby reducing the survival rate.

## 1. Introduction

According to the World Health Organization, in 2014, approximately 1.9 billion adults were overweight, and at least 600 million adults were obese. In addition, 42 million children under the age of five were reported to be overweight or obese in 2013. Thus, obesity rates are expected to increase globally. Wolin and colleagues estimated that overweight and obesity cause approximately 20% of all cancer cases [1]. Epidemiological studies indicated that obesity is associated with increased risks of several types of cancer, such as colon, breast, and hepatic cancer [2,3].

Prostate cancer is the second leading cause of death from cancer in men in the U.S. [4]. However, studies assessing the link between prostate cancer and obesity have generated puzzling results. A recent review of the epidemiological data linking prostate cancer and obesity indicates that obesity is associated with reduced risk of nonaggressive, indolent disease, and increased risk of more aggressive disease with increased local and distant invasion. Obesity is also associated with worse post-treatment results and increased risk of prostate cancer death [5].

Similar to the results of epidemiological studies, there are inconsistent results among studies using mouse tumor models. Consumption of a Western-type diet (enriched in fat and cholesterol) accelerated tumor progression in the transgenic adenocarcinoma mouse prostate (TRAMP) model [6], and lowering dietary fat (corn oil) delays prostate cancer development in the Hi-Myc transgenic mouse model [7]. However, a recent study has shown that a high-fat diet (HFD) containing soybean oil increased tumor weight and tumor volume in TRAMP-C2 allograft tumor model but did not affect tumor development in the TRAMP model [8]. Therefore, more studies are needed to clarify the relationship between obesity and prostate cancer, and to explore the underlying mechanisms.

Numerous studies have reported the biological mechanisms linking obesity to cancer, including: insulin and insulin-like growth factor, sex steroids, adipokines, inflammation, and obesity-induced hypoxia [9,10,11]. Adipose tissue, which is made up of various cell types (including adipocytes, fibroblasts, macrophages, and blood vessels), serves as an important endocrine organ and secretes adipokines (leptin, tumor necrosis factor, interleukin (IL)-6, monocyte chemoattractant protein (MCP)-1, vascular endothelial growth factor (VEGF), *etc*.). The well-known adipokines leptin and adiponectin were associated with cancer [12,13]. In addition, some adipokines (for example, leptin and MCP-1) contribute to the accumulation of macrophages in adipose tissue [14,15,16]. Therefore, obesity is thought to induce a state of chronic low-grade inflammation. Because chronic inflammation has been linked to various steps involved in tumorigenesis, including initiation, promotion, malignant conversion, invasion, and metastasis [17], obesity-induced inflammation and various adipokines may play an important role in the development and progression of prostate cancer via endocrine mechanisms. In addition, the results from recent studies suggested that cancer-associated adipocytes may also stimulate tumor progression via paracrine mechanisms [18,19].

In the present study, we examined the effects of a high-fat diet (HFD) containing lard on tumor development and progression using the TRAMP and TRAMP-C2 allograft models. In TRAMP mice, prostate cancer spontaneously develops with subsequent progression to metastasis. We also attempted to examine the effects of HFD on the survival rate of TRAMP mice, and to explore the pro-tumorigenic roles of soluble factors released from the adipose tissue of HFD-fed mice. Since the major cause of obesity is considered to be consumption of a Western-type diet, rich in animal fat and saturated fat, an animal fat (lard)-enriched diet (Table 1) was employed in the present study.

**Table 1:** Compositions of experimental diets.

	Control Diet (10 kcal % Fat)		High-Fat Diet (60 kcal % Fat)	
Product #	D12450B		D12492	
Ingredient	g	kcal	g	kcal
Casein, 80 Mesh	200	800	200	800
l-Cystine	3	12	3	12
Corn starch	315	1260	0	0
Maltodextrin 10	35	140	125	500
Sucrose	350	1400	68.8	275.2
Cellulose, BW200	50	0	50	0
Soybean Oil	25	225	25	225
Lard *	20	180	245	2205
Mineral Mix S10026	10	0	10	0
Dicalcium Phosphate	13	0	13	0
Calcium Carbonate	5.5	0	5.5	0
Potassium Citrate, 1 H_2_O	16.5	0	16.5	0
Vitamin Mix V10001	10	40	10	40
Choline Bitartrate	2	0	2	0
FD & C Yellow Dye #5	0.05	0		
FD & C Blue Dye #1			0.05	0
Total	1055.05	4057	773.85	4057

* Typical analysis of cholesterol in lard = 0.95 mg/g.

## 2. Experimental Section

### 2.1. Reagents

The following reagents were purchased from the indicated suppliers: 3-(4,5-dimethylthiazol-2-yl)-2,5-diphenyltetrazolium bromide (MTT), Sigma (St. Louis, MO, USA); Matrigel, BD Biosciences (San Jose, CA, USA); antibodies against proliferating cell nuclear antigen (PCNA), cyclin-dependent kinase (CDK)2, CDK4, Cyclin A, CD31, VEGF-A, and VEGF-C, Santa Cruz Biotechnology (Santa Cruz, CA, USA); antibodies against Ki67, VEGF-D, and lymphatic vessel endothelial hyaluronan receptor (LYVE)-1, Abcam (Cambridge, MA, UK); anti-VEGF receptor (VEGFR)-2, Cell Signaling (Beverly, MA, USA); antibodies against CD45 and CXCR5, recombinant mouse MCP-1, CXCL1, CXCL2 and CXCL13 protein, R & D systems (Minneapolis, MN, USA).

### 2.2. Cell Culture

TRAMP-C2 cells (established from a prostate tumor of a TRAMP mouse) [20] were purchased from the American Type Culture Collection (Manassas, MA, USA) and maintained in Dulbecco’s Modified Eagle’s Medium (DMEM) containing 10% fetal bovine serum (FBS), 100,000 U/L penicillin, and 100 mg/L streptomycin. Human umbilical vein endothelial cells (HUVECs) were purchased from Lonza (Walkersville, MD, USA) and maintained in Medium 199 (M199) containing 20% FBS, 100,000 U/L penicillin, 100 mg/L streptomycin, 1.5 μg/L epidermal growth factor, and 17 μg/L hydrocortisone. For all *in vitro* experiments, the cells were subjected to no more than 10 cell passages.

### 2.3. Animal Studies

#### 2.3.1. TRAMP-C2 Allograft Tumor Model

Four-week old, male C57BL/6J mice (Jackson Laboratory, Bar Harbor, ME, USA) were fed a commercially semi-purified control diet (CD, 10 kcal % fat, catalogue #D12450B, Research Diets Inc., New Brunswick, NJ, USA) or a high-fat diet (HFD, 60 kcal % fat, D12492, Research Diets, Inc.) *ad libitum* (10 mice/group). Twenty-four weeks after the beginning of feeding, TRAMP-C2 cells (1 × 10^6^ cells suspended in 0.1 mL Matrigel/PBS) were subcutaneously injected into the right rear flanks of the mice. All animals were killed 11 weeks after the TRAMP-C2 cell injection.

#### 2.3.2. TRAMP Model

TRAMP mice (Jackson Laboratory) were bred and maintained under specific pathogen-free conditions at the animal facility of Hallym University (Chuncheon, Korea). After selection of male TRAMP mice by genotyping [21], male TRAMP mice and their nontransgenic littermates at 4 weeks of age were fed the CD or the HFD *ad libitum*. In order to evaluate the effects of HFD on the survival rate of TRAMP mice, mice were fed a CD (*n* = 21) or a HFD (*n* = 24) for up to 46 weeks (50 weeks of age). To avoid pain or distress to the mice, we followed the guidelines for the use of animals in the cancer research published in the British Journal of Cancer [22]. When symptoms including severe body weight loss, persistent hypothermia, hunching behavior, *etc.*, were noted in a mouse, the mouse was euthanized with CO_2_ asphyxiation. Euthanasia was then equated with death.

To evaluate the effects of HFD on prostate cancer development and metastasis, mice were sacrificed at 24 and 32 weeks of age, respectively. (24 weeks: CD- and HFD-fed nontransgenic littermates, *n* = 6 mice/group; CD- and HFD-fed TRAMP, *n* = 12 mice/group and 32 weeks: CD- and HFD-fed nontransgenic littermates, *n* = 8 mice/group; CD-fed TRAMP, *n* = 17 mice; HFD-fed TRAMP, *n* = 20 mice).

At the time of sacrifice, mice were anesthetized with an intraperitoneal injection of 2.5% Avertin, and blood was harvested by retro-orbital bleeding, and the serum was stored at −70 °C for further analysis. The tumor (from TRAMP-C2 allograft tumor model), genitourinary (GU) tract (from TRAMP model), liver, lung, and fat tissues were removed, weighed, and fixed in 4% paraformaldehyde. Hemoglobin contents in tumor tissues were determined using Drabkin’s solution and a cyanmethemoglobin standard solution (Sigma) as described previously [23]. All animal experiments were approved by the Animal Care and Use Committee of Hallym University (Hallym2009-124 and Hallym2009-125).

### 2.4. Immunohistochemical (IHC) and Immunofluorescence (IF) Analysis

Fixed tissue samples were embedded in paraffin, and 5 μm sections were prepared. For the evaluation of pathologic grades in the dorsolateral lobes of the prostate, the paraffin-embedded sections were stained with hematoxylin and eosin (H & E). For IHC analyses, the paraffin-embedded sections were incubated with their relevant antibodies, and then developed using an LSAB^+^ kit (Dako, Carpinteria, CA, USA) in accordance with the manufacturer’s instructions.

For IF staining, the tumor sections were incubated with their relevant antibodies, and then incubated with corresponding secondary antibodies labeled with Alexa Fluor 488 (Invitrogen, Carlsbad, CA, USA) or Cy3 (Rockland, Gilbertsville, PA, USA). Nuclei were counterstained with 4’,6-diamidino-2-phenylindole (DAPI).

### 2.5. Preparation of Adipose Tissue-Conditioned Media (ATCM)

ATCM were prepared as described previously [23]. Briefly, chopped epididymal fat tissue from the CD- or HFD-fed C57BL/6 mice (16 weeks of diet feeding) was incubated in serum-free DMEM/M199 (1:1) supplemented with 50 μg/mL gentamicin and 0.5 μg/mL amphotericin B (serum-free medium, SFM). After 24 h, ATCM were collected for *in vitro* experiments.

### 2.6. Cytokine Array and Enzyme Linked Immunosorbent Assay (ELISA)

The levels of cytokines in pooled samples (serum (normal, 6 mice/group; TRAMP, 12 mice/group) or ATCM (5 mice/group)) were measured using Proteome Profiler™ (mouse cytokine array) in accordance with the manufacturer’s instructions (R&D Systems). The relative abundance of each dot to positive control spots (contained within the membrane) was quantified by densitometric scanning of the exposed film using Image J (NIH, Bethesda, MD, USA). The concentrations of MCP-1, IL-6, CXCL1, CXCL2, and CXCL13 were measured using ELISA kits in accordance with the manufacturer’s instructions (R & D Systems).

### 2.7. MTT Assay and Western Blot Analysis

TRAMP-C2 cells were plated in 24-well plates at 50,000 cells/well with DMEM containing 10% FBS. One day later, cells were serum-deprived for 24 h in DMEM containing 1% FBS. The cells were then treated with 0 or 50% ATCM for 24 h. Viable cell numbers were estimated by the MTT assay. Total cell lysates were prepared and Western blot analysis was performed as previously described [24].

### 2.8. Transwell Migration Assay

Transwell migration assay was conducted as described [25]. Briefly, TRAMP-C2 cells were added to the upper chamber, and the lower chamber of the well was filled with SFM in the absence or presence of 50% ATCM or 100 μg/L recombinant proteins (MCP-1, CXCL1, and CXCL2). Cells were then incubated for 1 h. Migrated cells into Type IV collagen-coated membrane were stained with H&E and counted. In order to examine the effect of CXCL13-CXCR5 axis on cell migration, TRAMP-C2 cells were pretreated with CXCR5 antibody (1 mg/L) for 30 min and CXCL13 (100 μg/L) was added to the lower chamber.

### 2.9. Tube Formation Assay and Aorta Ring Assay

HUVECs (50,000 cells) were plated in Matrigel-coated 24-well plates and incubated with SFM in the absence or presence of 5% ATCM. Tubular structures were photographed and tube length was quantified. For *ex vivo* aorta ring assay, aortic rings (1 mm thickness) taken from thoracic aortas of Sprague Dawley rats (24 weeks of age, Orient Bio Inc, Gapyung, Korea) were seeded in Matrigel-coated 48-well plates and treated with SFM in the absence or presence of 5% ATCM. Aortic rings were photographed using a light microscope.

### 2.10. Statistical Analysis

The results were expressed as means ± SEM. Differences between the two groups were assessed by Student’s *t*-test. When there were more than three groups, differences were tested by ANOVA followed by Duncan’s multiple range test, utilizing the SAS statistical software version 9.2 (SAS Institute, Cary, NC, USA). Survival rates were estimated from Kaplan-Meyer curves by log-rank test. Differences were considered significant at *p* < 0.05.

## 3. Results

### 3.1. HFD Feeding Increases Solid Tumor Growth in the TRAMP-C2 Allograft Model

**Figure 1:**
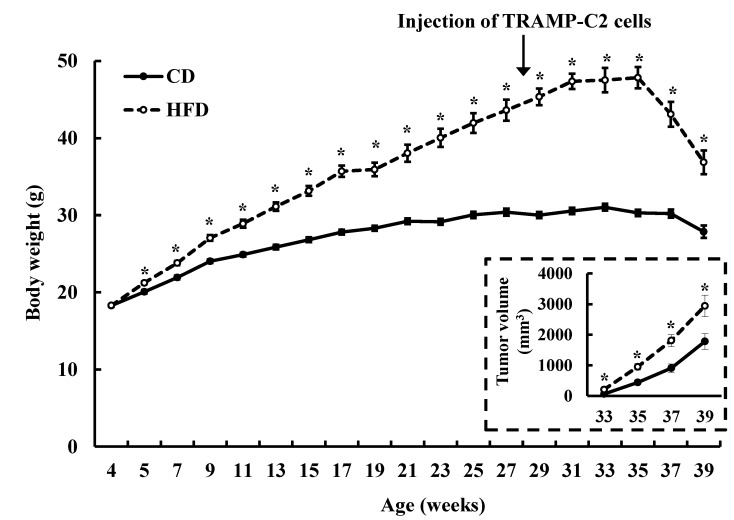
HFD feeding increases body weight in C57BL/6J mice injected with TRAMP-C2 cells. Four-week-old, male C57BL/6J mice were fed a control diet (CD, 10 kcal % fat) or high-fat diet (HFD, 60 kcal % fat) for a period of 24 weeks. At 28 weeks of age, TRAMP-C2 cells were subcutaneously injected into the right rear flanks of the mice. Eleven weeks after the TRAMP-C2 cell injection, all mice were sacrificed. Body weights of mice were measured every week. Results represent the means ± SEM (*n* = 10). The dotted box in the figure shows changes in tumor volume. * *p* < 0.05 as compared with the CD group.

In order to induce obesity, 4-week old male C57BL/6J mice were fed a HFD for 24 weeks until 28 weeks of age. Daily caloric intakes (kcal/day) were 10.59 ± 0.06 and 13.00 ± 0.17 in the CD and HFD groups, respectively (*p* < 0.001). The HFD group showed an increase in body weight compared to the CD group. Syngeneic TRAMP-C2 cells were then subcutaneously injected into the flanks of the mice, and the mice were sacrificed 11 weeks after the injection of TRAMP-C2 cells. Body weights of the HFD-fed mice were further increased until 35 weeks of age, after which they started to decrease as the tumor size increased. Body weights of the HFD-fed mice decreased by 24% (*p* = 0.0001) between 35 and 39 weeks of age. However, the body weights of CD-fed mice showed only an 8% decrease (*p* = 0.0065) during this period (Figure 1). During this period, rapid increase of the tumor volumes occurred. This increase was much greater in the HFD group (Figure 1 insert). At the time of sacrifice (39 weeks of age), the weights of the body, liver and fat tissues (epididymal and mesenteric fat), as well as of the tumor, were all higher in the HFD group of the TRAMP-C2 allograft model (Table 2).

**Table 2:** The body weights and organ weights of mice injected with TRAMP-C2 cells.

		Body	Liver	Tumor	Epididymal Fat	Mesenteric Fat
CD	(g)	27.87 ± 0.80	1.16 ± 0.04	1.54 ± 0.21	0.50 ± 0.06	0.10 ± 0.02
	(% of BW)		4.18 ± 0.12	5.45 ± 0.69	1.71 ± 0.18	0.35 ± 0.06
HFD	(g)	36.87 ± 1.53 *	1.55 ± 0.06 *	2.60 ± 0.29 *	1.01 ± 0.12 *	0.38 ± 0.07 *
	(% of BW)		4.30 ± 0.18	7.21 ± 0.78	2.58 ± 0.21 *	0.90 ± 0.15 *

Four-week-old, male C57BL/6J mice were fed a control diet (CD, 10 kcal % fat) or high-fat diet (HFD, 60 kcal % fat) for a period of 24 weeks. After 24 weeks, TRAMP-C2 cells were subcutaneously injected into the right rear flanks of the mice. Eleven weeks after the TRAMP-C2 cell injection, all mice were sacrificed. At the time of sacrifice, body weights and organ weights were measured. * *p* < 0.05 as compared with the CD group. BW, body weight.

**Figure 2:**
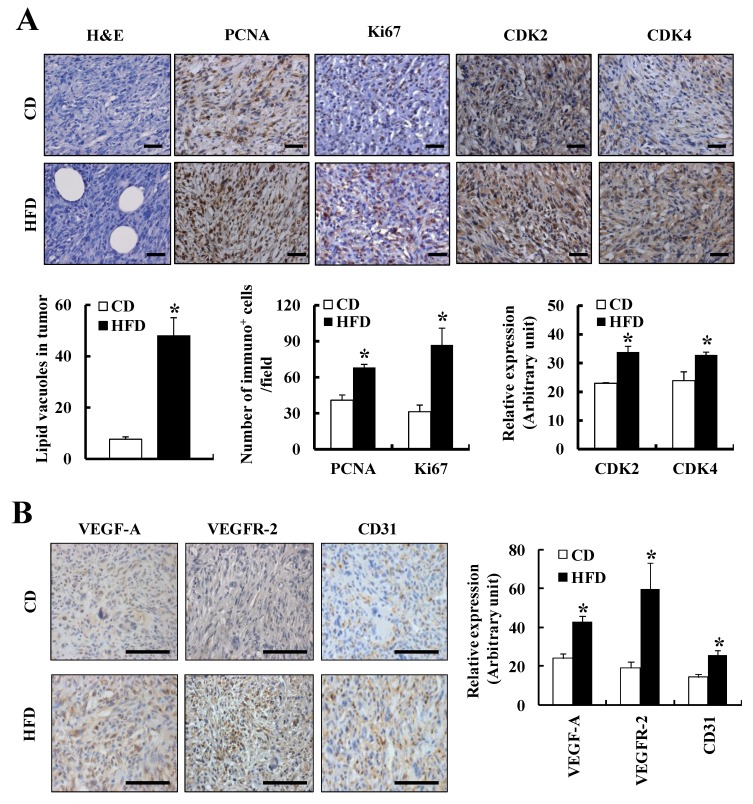

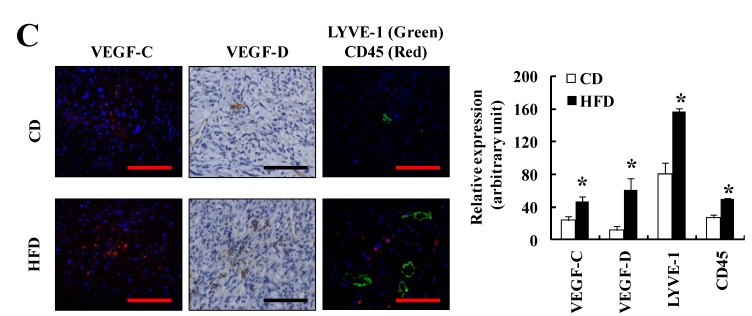
HFD feeding increases tumor proliferation, angiogenesis, and lymphangiogenesis in the TRAMP-C2 allograft model. Four-week-old, male C57BL/6J mice were fed a CD or HFD for a period of 24 weeks. After 24 weeks, TRAMP-C2 cells were subcutaneously injected into the right rear flanks of the mice. Eleven weeks after the TRAMP-C2 cell injection, all mice were sacrificed and tumors were isolated. Tumor sections were stained with the indicated antibodies, and nuclei were counterstained with hematoxylin or DAPI. (**A**) Representative images of IHC staining for proliferation-related proteins (upper panel) and quantification results (lower panel) are shown; (**B**) Representative images of IHC staining for angiogenesis-related proteins (left panel) and quantification results (right panel) are shown; (**C**) Representative images of IHC staining for lymphangiogenesis-related proteins (left panel) and quantification results (right panel) are shown. Each bar represents the mean ± SEM (*n* = 8). * *p* < 0.05 as compared with the CD-fed group. Scale bar, 100 μm.

The IHC analysis results revealed that the number of lipid vacuoles and the expression of PCNA, Ki67, CDK2, and CDK4 were significantly greater in tumor tissues obtained from the mice fed on the HFD as compared to those of CD-fed mice (Figure 2A). However, the number of TUNEL^+^ apoptotic cells in tumor tissues was not affected by HFD feeding (data not shown). Additionally, HFD feeding significantly increased the expression of proteins related to angiogenesis (VEGF-A, VEGFR-2, and CD31) (Figure 2B) and lymphangiogenesis (VEGF-C, VEGF-D, and LYVE-1) (Figure 2C) in tumor tissues. Furthermore, hemoglobin contents (mg/g tissue) in tumor tissues were 23.9 ± 3.0 and 45.6 ± 4.9 in the CD and HFD group (*p* < 0.05), respectively. These results indicate that HFD increases solid tumor growth, angiogenesis, and lymphangiogenesis in the TRAMP-C2 allograft model. However, neither lung nor liver metastasis was detected when the mice were sacrificed at 11 weeks after the tumor cell injection in this allograft model.

### 3.2. HFD Feeding Increases Prostate Cancer Development and Progression in TRAMP Mice

In order to evaluate the effects of HFD on prostate cancer development and progression, TRAMP mice and their nontransgenic littermates (normal mice) were fed the CD or the HFD, and mice were sacrificed at 24 or 32 weeks of age. As expected, the daily calorie intakes (kcal/day) of the HFD groups were significantly higher than those of the CD groups (*p* < 0.001) (normal mice: CD-fed mice, 9.56 ± 0.08; HFD-fed mice, 11.43 ± 0.09; TRAMP: CD-fed TRAMP, 9.08 ± 0.07; HFD-fed TRAMP, 11.14 ± 0.09). At 24 weeks of age, the weights of body, liver, and fat tissues were substantially higher in HFD-fed TRAMP mice and normal littermates as compared to CD-fed TRAMP and normal mice (Table 3). As expected, the weight of GU tracts in TRAMP mice was increased as compared to that in normal littermates. In TRAMP mice, HFD feeding increased the GU tract weights significantly. HFD feeding tended to increase the GU tract weights of normal mice, but the difference was not statistically significant (Table 3). When mice were sacrificed at 32 weeks of age, the GU tract weights of HFD-fed normal mice were significantly higher than those of CD-fed normal mice, and those of TRAMP mice were increased remarkably by HFD feeding (Table 3).

**Table 3:** The body weights and organ weights (g) of TRAMP mice and their normal littermate controls.

	Body	Liver	GU Tract	Epididymal Fat	Mesenteric Fat
**TRAMP Model (24 Weeks of Age)**					
Normal mice-CD	28.49 ± 0.50	0.95 ± 0.11	0.37 ± 0.04	0.84 ± 0.13	0.23 ± 0.02
Normal mice-HFD	42.87 ± 1.73 *	2.08 ± 0.08 *	0.44 ± 0.02	2.54 ± 0.22 *	1.46 ± 0.05 *
TRAMP-CD	27.44 ± 0.40	0.97 ± 0.05	0.66 ± 0.06 *	0.73 ± 0.09	0.19 ± 0.03
TRAMP-HFD	41.42 ± 0.93 ^*f*^	1.95 ± 0.19 ^*f*^	0.93 ± 0.08 ^*f*^	2.16 ± 0.15 ^*f*^	1.10 ± 0.10 ^*f*^
**TRAMP Model (32 Weeks of Age)**					
Normal mice-CD	31.19 ± 1.13	1.20 ± 0.08	0.53 ± 0.03	1.10 ± 0.18	ND
Normal mice-HFD	48.90 ± 1.63 *	1.90 ± 0.22 *	0.73 ± 0.09 *	1.80 ± 0.20 *	ND
TRAMP-CD	27.67 ± 0.49	1.04 ± 0.04	0.87 ± 0.08 *	0.63 ± 0.05	ND
TRAMP-HFD	41.60 ± 2.25 ^*f*^	1.67 ± 0.17 ^*f*^	1.99 ± 0.16 ^*f*^	1.79 ± 0.18 ^*f*^	ND

Male TRAMP mice and their nontransgenic littermates at four weeks of age were fed a CD or HFD for 20 or 28 weeks. At the time of sacrifice, body weights and organ weights were measured. * *p* < 0.05 as compared with the CD-fed normal mice group. ^*f*^ *p* < 0.05 as compared with the CD-fed TRAMP group. GU tract, genitourinary tract. ND, not determined.

**Figure 3:**
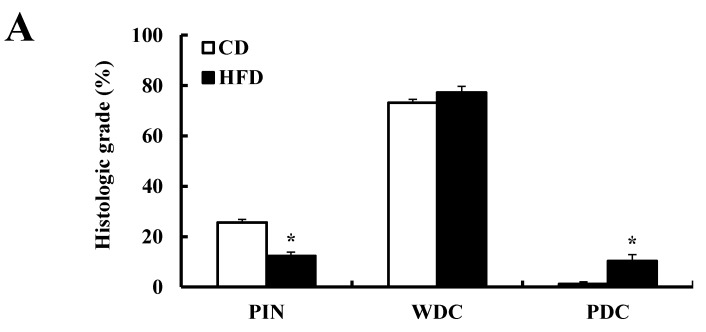

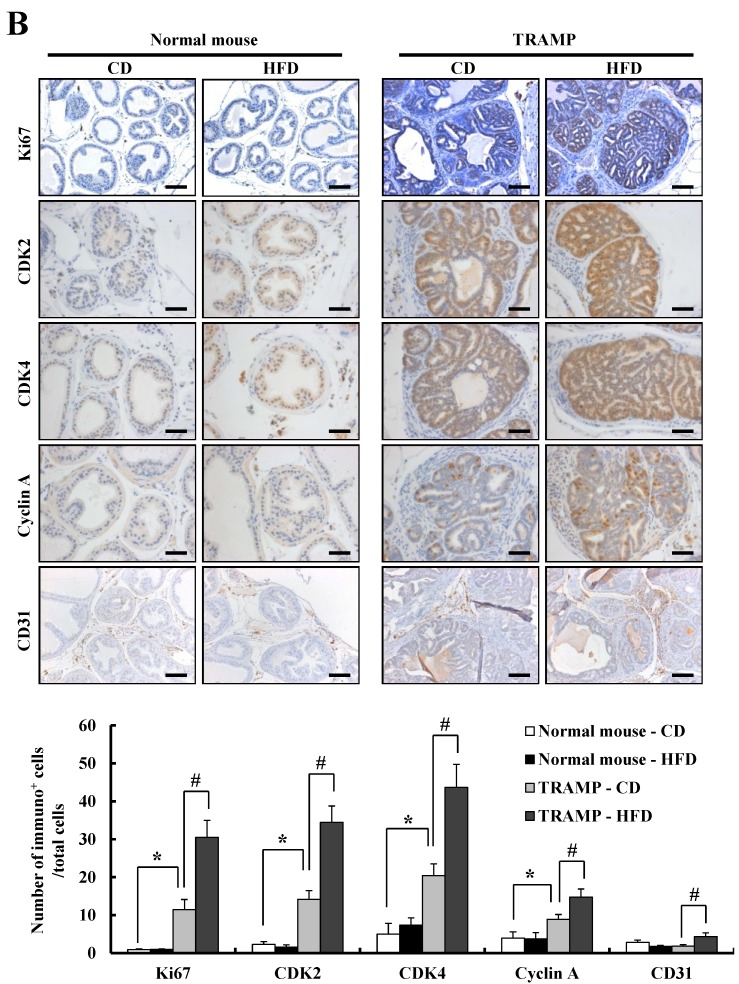
HFD feeding increases prostate cancer development in TRAMP mice. Male TRAMP mice and their nontransgenic littermates at four weeks of age were fed a CD or HFD for a period of 20 weeks. All mice were sacrificed at 24 weeks of age, and the prostate was removed and fixed in 4% paraformaldehyde. (**A**) Prostate sections were stained with H&E. Each bar represents the percentage (mean ± SEM) of each pathologic grade in the dorsolateral lobes of the prostate (DP) of TRAMP mice (*n* = 12). * *p* < 0.05 as compared with the CD-fed TRAMP group. PIN, prostatic intraepithelial neoplasia; WDC, well-differentiated carcinoma; PDC, poorly-differentiated carcinoma; (**B**) Prostate sections were stained with the indicated antibodies, and nuclei were counterstained with hematoxylin. Representative images of IHC staining (upper panel) and quantification results (lower panel) are shown. Each bar represents the mean ± SEM (*n* = 6 (normal mouse) or 8 (TRAMP)). * *p* < 0.05 as compared with the CD-fed normal mice group. ^#^ *p* < 0.05 as compared with the CD-fed TRAMP group. Scale bar, 100 μm.

H & E staining of the dorsolateral lobes of the prostate (DP) revealed that well-differentiated carcinoma (WDC) was the predominant pathology at 24 weeks of age in both CD- and HFD-fed TRAMP mice. There were no differences in the proportion of lobes with WDC between the two groups at this time. On the other hand, the number of lobes with prostatic intraepithelial neoplasia (PIN) was lower, and that of lobes with poorly-differentiated carcinoma (PDC) was higher in the HFD-fed mice compared to the CD-fed mice (Figure 3A), suggesting that HFD feeding accelerates the development of prostate cancer. We next investigated the effects of HFD on the expression of proliferation-related molecules via IHC. Ki67^+^ cells, as well as the expression of CDK2, CDK4, and Cyclin A, were increased in the DP with WDC of TRAMP mice in comparison with normal littermates. In addition, these increases were further augmented by HFD feeding. These results indicate that HFD feeding stimulates the development of prostate cancer through the induction of cell cycle progression, thereby speeding up the progression to the PDC state in TRAMP mice. Moreover, we observed that cells positive for CD31, a blood endothelial cell marker, were increased in the prostate of HFD-fed TRAMP mice (Figure 3B).

### 3.3. HFD Feeding Reduces Survival Rate in TRAMP Mice

**Figure 4:**
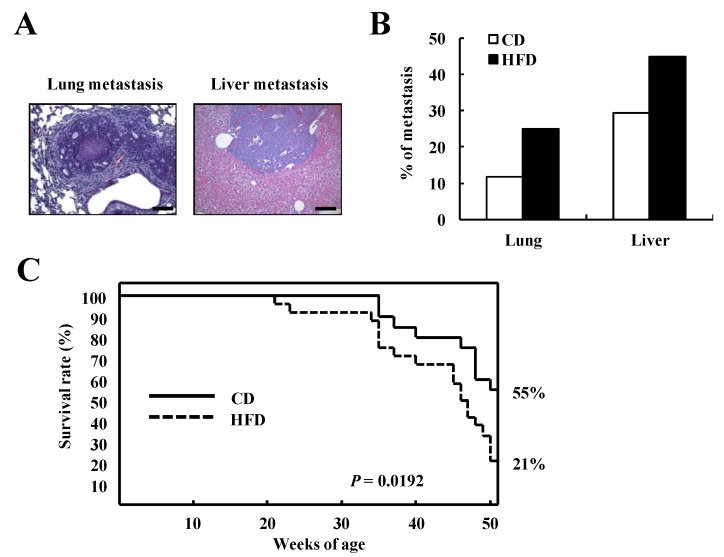
HFD feeding reduces the survival rate in TRAMP mice. (**A**, **B**) TRAMP mice were fed a CD (17 mice) or HFD (20 mice) for a period of 28 weeks. Mice were sacrificed at 32 weeks of age, the lungs and livers were removed, and the lung and liver sections were stained with H&E; (**A**) Representative images; (**B**) Incidence of metastasis. Each bar represents the percentage of mice having metastatic lesions; (**C**) TRAMP mice were fed the CD (21 mice) or the HFD (24 mice). Survival rates of TRAMP mice were estimated until the mice were 50 weeks of age. Scale bar, 100 μm.

We next investigated the effect of HFD on metastasis in TRAMP mice at 32 weeks of age. The incidence of lung and liver metastasis in CD-fed TRAMP mice was 11.8% and 29.4%, respectively. HFD feeding increased the incidence of lung (25%) and liver (45%) metastasis (Figure 4A,B). In order to estimate the effects of HFD on the survival rate in TRAMP mice, TRAMP mice were fed the CD or HFD up to 50 weeks of age. At 50 weeks of age, the survival rate of CD-fed TRAMP mice was 55%, whereas that of the HFD-fed mice was 21% (*p* = 0.0192 by the log-rank test, Figure 4C).

### 3.4. HFD Feeding Induces Changes in Serum Cytokine Levels

**Figure 5:**
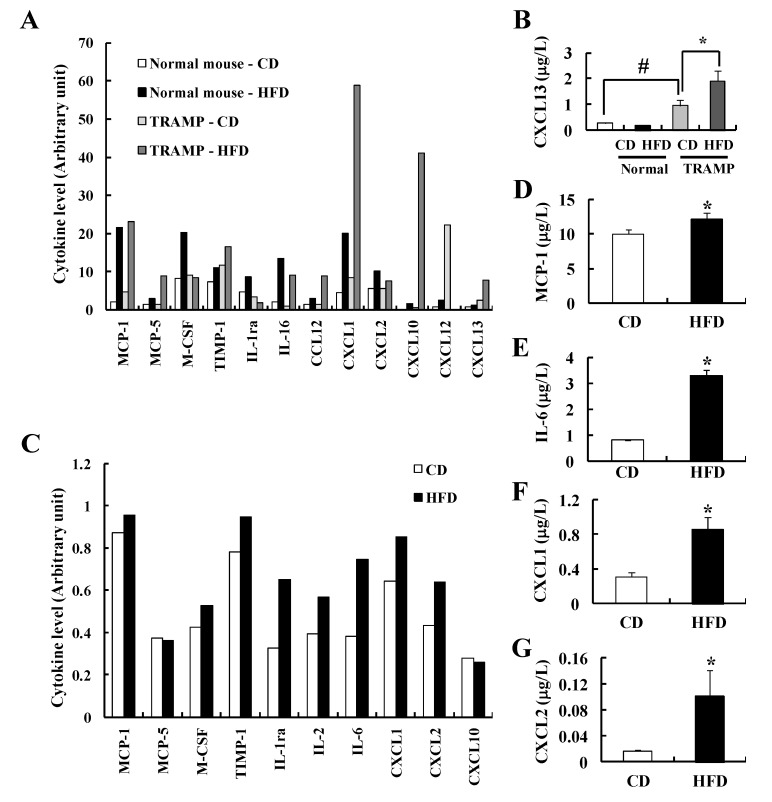
HFD feeding induces the changes in cytokine levels. (**A**, **B**) Four-week-old TRAMP mice and their normal littermates were fed a CD or HFD for 20 weeks and sera were prepared; (**C**–**G**) Adipose tissue conditioned media (ACTM) were prepared from 20-week-old mice that had been fed the CD or HFD for 16 weeks. The relative levels of cytokines in the pooled serum samples (normal, 6 mice/group; TRAMP, 12 mice/group) (**A**) and ATCM (5 mice/group) (**C**) were estimated by Proteome Profiler™ (mouse cytokine array) as described in the Materials and Methods section; (**B**, **D**–**G**) The concentrations of CXCL13 in serum (**B**) and the concentrations of MCP-1, IL-6, CXCL1, and CXCL2 in ATCM (**D**–**G**) were determined by ELISA. Each bar represents the mean ± SEM. ^#^ *p* < 0.05 between normal mice and TRAMP mice. * *p* < 0.05 as compared with the CD-fed group.

Because adipose tissue secretes a wide variety of growth factors and cytokines that were associated with tumor progression, we have employed cytokine arrays to identify alterations by HFD feeding in the current animal models. We noted that the levels of MCP-1, M-CSF, IL-1ra, IL-16, CXCL1, CXCL2, and CXCL10 were increased in the sera of HFD-fed nontransgenic normal mice compared to CD-fed normal mice (Figure 5A). In TRAMP mice, HFD feeding increased the serum levels of MCP-1, MCP-5, TIMP-1, IL-16, CCL12, CXCL1, CXCL10, and CXCL13. Similar results were observed in the sera of the TRAMP-C2 allograft model (data not shown). The levels of CXCL1, CXCL10, and CXCL13 were elevated in the sera of HFD-fed TRAMP mice as compared to HFD-fed normal mice (Figure 5A). ELISA result revealed that serum CXCL13 concentrations were increased in CD-fed TRAMP mice as compared to those in CD-fed normal mice, which were further increased in HFD-fed TRAMP mice (Figure 5B). In order to examine the changes of cytokine production in adipose tissue, we prepared ATCM from epididymal fat tissues of normal mice [23]. The levels of MCP-1, M-CSF, IL-1ra, IL-6, CXCL1, and CXCL2 were increased in ATCM obtained from HFD-fed mice (Figure 5C–G), suggesting that some cytokines produced by adipose tissue leaks into the circulation and affects the blood cytokine profiles.

### 3.5. Soluble Factors Released by Adipose Tissues Stimulate Tumor Progression

**Figure 6:**
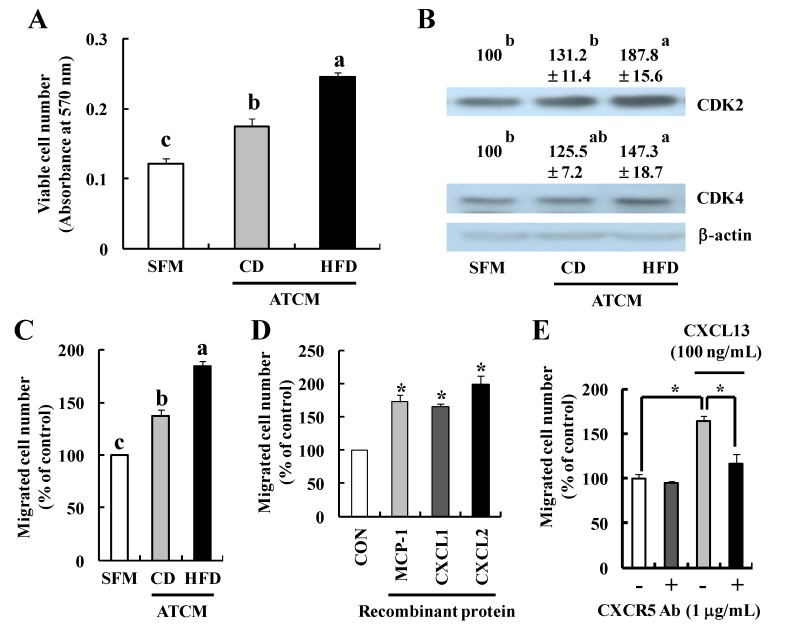

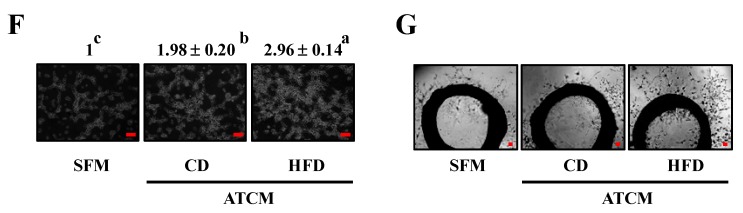
Adipose tissue-conditioned media (ATCM) from high-fat diet-fed mice stimulate the proliferation and migration of prostate cancer cells and angiogenesis. (**A**) TRAMP-C2 cells were treated with 0 or 50% ATCM for 24 h. Cell viability was determined by MTT assay. Each bar represents the mean ± SEM (*n* = 3); (**B**) Total cell lysates were prepared and analyzed via Western blot analysis with the indicated antibodies. The relative abundance of each band to its own β-actin was quantified and the control levels were set at 100%. The adjusted mean ± SEM (*n* = 3) is shown above each blot; (**C**–**E**) The migration of TRAMP-C2 cells through a type IV collagen-coated transwell filter was assessed. Fifty percent ATCM (**C**) or 100 μg/L recombinant proteins (**D**) were added to the lower chamber of the transwell as a chemoattractant; (**E**) CXCL13 (100 μg/L) was added to the lower chamber and TRAMP-C2 cells were pretreated with CXCR5 antibody (1 mg/L). Each bar represents the mean ± SEM (*n* = 3). (**F**) HUVEC tube formation assay. Cumulative tube length was quantified and shown above each photograph. The control levels were set at 1. Each value represents the mean ± SEM (*n* = 3); (**G**) Aorta ring assay. The photographs, which are representative of three independent experiments, are shown; (**A**–**C** and **F**) Means without the same letter (a, b, or c) are significantly different (*p* < 0.05); (**D**) * *p* < 0.05 as compared with the control group; (E) * *p* < 0.05 between the two groups. Scale bar, 100 μm.

In order to examine whether soluble factors produced by adipose tissues stimulate tumor cell proliferation and migration and/or angiogenesis, we conducted *in vitro* studies using ATCM. ATCM from both CD- and HFD-fed mice increased the proliferation of TRAMP-C2 cells, and ATCM from HFD-fed mice were more effective than that from CD-fed mice (Figure 6A). ATCM from HFD-fed mice increased CDK2 and CDK4 expression in TRAMP-C2 cells (Figure 6B). Moreover, ATCM from HFD-fed mice were more effective in stimulating migration of TRAMP-C2 cells (Figure 6C). Cytokines (MCP-1, CXCL1, and CXCL2), which were elevated in sera and ATCM of HFD-fed mice (Figure 5D,F,G), increased TRAMP-C2 cell migration (Figure 6D). We also observed that CXCL13, which was increased in the sera of HFD-fed TRAMP mice (Figure 5B), stimulated TRAMP-C2 cell migration and this increase in migration was attenuated by a CXCR5 neutralizing antibody (Figure 6E). In addition, ATCM from HFD-fed mice increased capillary-like tube formation of HUVECs (Figure 6F) and microvessel outgrowth from the rat aorta (Figure 6G) as compared to that from CD-fed mice. These results indicate that HFD feeding increases the production of soluble factors in adipose tissue, which stimulates the proliferation and migration of prostate cancer cells as well as angiogenesis by paracrine and endocrine mechanisms.

## 4. Discussion

Epidemiological studies assessing the link between prostate cancer and obesity have generated puzzling results. For example, even though several prospective cohort studies observed that obesity was associated with increased risk of death due to prostate cancer [26,27], large prospective cohort studies in the United States found that obesity was associated with a reduced diagnosis of prostate cancer [28,29,30]. Obese men have lower prostate-specific antigen (PSA) values than non-obese men, possibly due to hemodilution with larger volume in the obese men [31]. As a result, obese men have lower chances of having elevated PSA, receive less recommendation to undergo biopsy, and are less likely to be diagnosed with prostate cancer. In addition, obese men have larger prostates [32], making cancer detection more difficult at biopsy. Freedland *et al*., reported that obesity was associated with a 98% increased risk of prostate cancer after adjusting for the lower PSA levels and larger prostate size [33]. Using TRAMP mice, Llaverias *et al*., demonstrated that a Western-type diet containing high cholesterol and fat accelerates tumor progression [6]. Additionally, similar results were obtained from the Hi-Myc transgenic mouse model [7]. In the present study, we showed that feeding TRAMP mice with HFD containing lard induces body weight gain, and enhances tumor growth and progression, thereby leading to decrease in survival rate. Taken together, these results indicate that a HFD containing lard is detrimental for individuals who are genetically predisposed to prostate cancer, or have indolent prostate cancer.

In the present study, mice fed the HFD containing lard consumed more energy and also became obese. Epidemiological evidence indicates that obesity (elevated BMI) is positively correlated with prostate cancer-specific mortality [34,35]. Therefore, increased body weight can be a contributing factor to the increased tumor growth and high mortality observed in the mice fed lard. Dietary fat itself can also stimulate tumor progression. It has been reported that LNCaP tumor growth was decreased in nude mice when their diet was switched from a high-corn oil diet to a low fat diet (LFD) with no differences in total energy intakes or body weight gains [36]. Severe combined immunodeficient mice fed an isocaloric LFD diet displayed delayed LAPC-4 xenograft tumor growth and decreased blood PSA levels relative to mice fed a HFD containing corn oil [37]. Additionally, in a similar mouse model, reduction in dietary corn oil without changes in calorie intakes slowed down the advancement of LAPC-4 xenografts from androgen dependency to independency [38]. These results clearly indicate that dietary fat, without changes in energy intake or body weight, stimulates tumor promotion. The present study did not determine whether the dietary fat or obesity itself was responsible for the stimulation of tumor progression and reduction in survival rates in these animal models. However, in the allograft models, we noted that the body weights of the tumor-bearing mice started to decrease when the tumor volumes increased very rapidly, which was more pronounced in the HFD-fed mice (Figure 1). These results indicate that more energy was stored in the HFD-fed mice, which was in turn used to support the rapid tumor growth.

In addition to amount of fat intake, the composition and quality of the fat also affect tumor progression. For example, it was reported that a diet containing 35% kcal fish oil (rich in ω-3 fatty acid) slowed tumor growth and improved survival in the LAPC-4 xenograft model, as compared to a diet containing the same amount of olive oil, corn oil, or lard/milk fat [39]. Similarly, diets containing high levels of walnuts, fish oil, or docosahexaenoic acid and eicosapentaenoic acid were found to have beneficial effects on prostate cancer risk and tumorigenesis [39,40,41,42]. On the other hand, normolipidic diets containing pork fat (7% lard) have cancer-promoting effects (increase in prostatic weight associated with epithelial hyperplasia and increased expression of AR and PPARγ) [43]. These results indicate that the quality of the fat (e.g., the relative amounts of ω-3 fatty acid, ω-6/ω-3 ratio, and saturated fatty acid), as well as the quantity, is an important factor for prostate cancer progression. In addition to fat, the intake of protein is also associated with prostate cancer risk. A high intake of dairy protein was associated with an increased risk of human prostate cancer [44], whereas restriction of dietary protein intake (7% kcal) inhibited tumor growth in mouse xenograft models of human prostate and breast cancer [45]. In our allograft model, the daily protein intakes (g/day) were 0.53 ± 0.01 and 0.65 ± 0.01 in the CD and high lard groups, respectively. Thus, in addition to lard, the increased protein intake may have been a factor in increased tumor progression in the mice fed the HFD containing lard.

Increased levels of a variety of cytokines were observed in the blood and adipose tissues of HFD-fed mice, and the changes in these cytokines were found to be associated with the stimulation of tumor progression. It has been reported that different types of dietary fat exert different effects on inflammatory responses [46,47] as well as cancer [48]. For example, saturated fats and n-6 polyunsaturated fats have pro-inflammatory properties and are associated with increased risk of prostate cancer, while n-3 polyunsaturated fats and monounsaturated fats are known to have anti-inflammatory and anti-cancer properties [46,47,48]. The HFD used in the present study contained more lard than the CD. Unfortunately, the present results cannot provide any information regarding whether the differences in cytokine production between the CD and HFD groups were due to the differences in lard intake in the diet, or simply due to the development of obesity itself. Future studies are needed to determine the effects of high-fat diet-induced obesity, as well as the effects of different types of fat on tumor progression and tumor-stimulating molecules, such as cytokine production.

Several hypotheses were proposed to explain the association between obesity and cancer. Of those, a recent study showed that adipocytes surrounding the tumor, which are referred to as cancer-associated adipocytes (CAAs), contribute to breast cancer invasion via modifying the cancer cell characteristics/phenotype [18]. Moreover, Nieman and colleagues suggest that adipocytes provide adipokines to attract tumor cells and fatty acids for tumor growth [19]. These studies suggest that adipose tissues provide fatty acids and soluble factors that stimulate tumor growth and metastasis via a paracrine mechanism. In addition, our previous results showed that adipocytes produce chemoattractants, which induce monocyte migration. We have also shown that the crosstalk between melanoma cells, adipocytes and macrophages increases chemoattractants for monocytes and angiogenic and lymphangiogenic factors [49]. In the present study, the number of lipid vacuoles and CD45^+^ leukocytes was increased in the tumor tissues of HFD-fed mice injected with TRAMP-C2 prostate cancer cells (Figure 2A,C). Taken together, these results indicate that HFD feeding increases CAAs that stimulate leukocyte infiltration into tumor tissues, and heterotypic interactions between CAAs, leukocytes, and tumor cells produce many growth factors, chemokines, and cytokines for tumor growth and progression.

In the present study, we observed that ATCM stimulated the growth and migration of prostate cancer cells and angiogenesis, which were further enhanced when ATCM were prepared from HFD-fed mice. Furthermore, ATCM from HFD-fed mice increased the expression of CDK2 and CDK4 in TRAMP-C2 cells (Figure 6). Consistent with these observations, several *in vitro* studies have shown that peri-prostatic adipose tissue explants secreted substances promoting proliferation and migration of prostate cancer cells [50,51]. We also noted that Ki67^+^ proliferating cells and the expression of cell proliferation-related proteins (CDK2, CDK4, and Cyclin A) were increased in the tumor tissues of HFD-fed mice injected with TRAMP-C2 prostate cancer cells (Figure 2) and in the DP of HFD-fed TRAMP mice (Figure 3). Taken together, these results indicate that soluble factors leaked from adipose tissues of HFD-fed mice stimulate prostate tumor progression via paracrine and/or endocrine mechanisms.

Serum adipokine levels are altered in obese animals and humans [52]. Consistent with this notion, in the blood of TRAMP mice, a variety of cytokines including MCP-1, CXCL1, CXCL10, CXCL13 *etc.* were markedly increased by HFD feeding. Similar changes were noted in nontransgenic littermates but the changes in some cytokines due to HFD feeding were smaller in the normal littermates (e.g., CXCL1 and CXCL10) (Figure 5A). We also noted that the levels of many of these cytokines were increased in the ATCM from HFD-fed nontransgenic C57BL/6J mice as compared to CD-fed mice (Figure 5C). These increases in cytokine levels indicate that adipose tissues in HFD-fed mice release increased levels of cytokines, and this increase substantially contributes to increases in serum cytokine levels because the weights of fat tissues were also tremendously increased in HFD-fed mice (Table 2 and Table 3).

In the present study, the levels of MCP-1, CXCL1, and CXCL2 were increased in the serum (Figure 5A) and ATCM (Figure 5C,D,F,G) of HFD-fed mice. TRAMP-C2 migration was increased by ATCM from HFD-fed mice as compared to that of CD-fed mice (Figure 6C), and cytokines induced by HFD feeding (MCP-1, CXCL1, and CXCL2) stimulates TRAMP-C2 cell migration (Figure 6D). It has been reported that MCP-1 levels both in the blood and in white adipose tissues are increased in HFD-fed mice and their blood levels correlate with changes in body weights [16,53]. MCP-1 induces the infiltration of macrophages into adipose tissues [16] as well as proliferation and invasion of prostate cancer cells [54]. CXCL1 and CXCL2 produce their effects by signaling through CXCR2 [55,56]. A study with human prostate biopsy reported that CXCR2 expression is correlated with advancing state of the disease [57]. Serum CXCL13 was significantly higher in prostate cancer patients compared to patients with benign prostatic hyperplasia or high-grade prostatic intraepithelial neoplasia and normal healthy donors, suggesting that CXCL13 is a better predictor of prostate cancer than PSA [58]. We observed that serum CXCL13 levels were increased in CD-fed TRAMP mice compared with those in CD-fed nontransgenic littermates, and the levels were further increased in HFD-fed TRAMP mice (Figure 5B). It has been also reported that CXCL13 increased migration and invasion of LNCaP and PC3 prostate cancer cells [59]. Expression of CXCR5, a corresponding receptor for CXCL13, was higher in prostate cancer cases, and the intensity of CXCR5 expression positively correlated with the Gleason score [60]. We also observed that CXCL13 stimulated TRAMP-C2 cell migration, which was attenuated by a CXCR5 neutralizing antibody (Figure 6E). Taken together, these results suggest that HFD-induced increases in the production of soluble factors (MCP-1, CXCL1, CXCL2, and CXCL13) play important roles in the stimulation of prostate tumor progression in mice.

## 5. Conclusions

In conclusion, the present study demonstrated that a HFD containing lard accelerates the development and progression of prostate cancer in mice, thereby reducing the survival rate. HFD feeding increased the solid tumor growth in the allograft model and stimulated the progression from PIN to PDC in the TRAMP model. HFD feeding also increased the size of the adipose tissues, as well as the levels of a variety of cytokines in the adipose tissue, and ATCM from HFD-fed mice stimulated the proliferation and migration of prostate cancer cells as well as angiogenesis. The present results suggest that increases in adipose tissue mass, including tumor-associated adipose tissues, and increases in adipose tissue-derived soluble factors in response to feeding of HFD contributes to the stimulation of cancer growth and metastatic potential in prostate cancer via endocrine and paracrine mechanisms.
